# 

**Published:** 1896-06-20

**Authors:** 


					the hospital. ABSOLUTELY PURE, therefore BEST,
June 20th, 1896.
" CADBURY'S CADBURY'S
icocoa <Hl\ Iri JfeT'1 cocoa
^ " PreSem ^ NO ALKALIES USED.
L? Eli _ _
AtcotT*wTsr^uTin^rm'aSy 1 -"Norfolk*mb Norwich hospi tal
No. 508>Voi. xx. WITH EXTRA NURSING SUPPLEMENT. I3KE?.1E!?1SK!B
S*TTrr,n.? Ttt,tt, Ofi^rr tone [Rsffifltered at the General Post Offloe as a Newspaper for Monthly Parts, 6d.; post free, 8d.
^SATURDAY, JUNE zUTH, 1 o;)b. tran*mi??ion in the United Kintrdn? and thrond/l  Dittorwr kirn-n^ nprt

				

